# How Arterial Embolization Is Transforming Treatment of Oncologic and Degenerative Musculoskeletal Disease

**DOI:** 10.3390/curroncol31120555

**Published:** 2024-11-26

**Authors:** Nicolas Papalexis, Giuliano Peta, Michela Carta, Simone Quarchioni, Maddalena Di Carlo, Marco Miceli, Giancarlo Facchini

**Affiliations:** Department of Diagnostic and Interventional Radiology, IRCCS-Istituto Ortopedico Rizzoli, 40136 Bologna, Italy; giuliano.peta@ior.it (G.P.); michela.carta@ior.it (M.C.); simone.quarchioni@ior.it (S.Q.); maddalena.dicarlo@ior.it (M.D.C.); marco.miceli@ior.it (M.M.); giancarlo.facchini@ior.it (G.F.)

**Keywords:** arterial embolization, therapeutic, musculoskeletal diseases, bone neoplasms, soft tissue neoplasms, palliative care, preoperative care, chronic pain, osteoarthritis

## Abstract

Background: Arterial embolization is a minimally invasive treatment that occludes blood vessels supplying pathological tissue. Developed to control bleeding without surgery, it has evolved over decades and is now applied in musculoskeletal oncology as a preoperative treatment, palliative care, or standalone therapy for select tumors. Recently, its use has expanded globally in treating chronic pain syndromes and osteoarthritis. Materials and Methods: We reviewed the literature on arterial embolization in various musculoskeletal conditions. The focus was on established oncologic indications for primary and metastatic bone or soft tissue tumors, and emerging evidence on degenerative diseases like osteoarthritis, inflammatory musculoskeletal pathology, and intractable pain. Emphasis was placed on leading studies regarding efficacy, complications, and recurrence rates. Discussion: Arterial embolization has progressed from bleeding control to a versatile therapeutic option in musculoskeletal medicine. It offers symptom relief, reduces tumor size, and improves quality of life. Applications include oncologic interventions and management of degenerative and inflammatory conditions. Despite its benefits, variations in complications and recurrence rates highlight the need for standardized protocols and further research. Conclusions: Arterial embolization is a safe and effective minimally invasive tool in the multidisciplinary management of a wide range of musculoskeletal pathologies. Ongoing research is crucial to understand long-term efficacy, optimize protocols, and broaden its applications.

## 1. Introduction

Arterial embolization has emerged as a crucial therapeutic tool within interventional radiology, offering a minimally invasive therapeutic option to numerous musculoskeletal pathologies. One of the first articles reporting the use of an endovascular approach as a therapeutic tool was published in 1964, for the treatment of atherosclerotic obstruction [1]. In the 1970s, the first articles started to appear on arterial embolization in the musculoskeletal system for traumatic bleeding control [2] and for the management of hypervascular tumors [3]. Since then, arterial embolization has become a key advanced medical procedure for the treatment of oncologic musculoskeletal pathology, and has also gained an increased prominence in the field of degenerative musculoskeletal diseases [4]. The procedure, which involves the selective endovascular occlusion of blood vessels supplying the areas of pathological tissue, combined with different embolic agents [4] including chemotherapeutic drugs, has significantly increased the therapeutic options for conditions ranging from bone metastases and benign tumors to chronic pain syndromes and osteoarthritis [5].

The routinary elective use of arterial embolization in musculoskeletal pathology first gained prominence in oncologic patients, especially for the management of bone metastases [6]. As metastatic disease is the most common malignancy affecting bone, effective palliation of symptoms such as intractable pain and pathologic fractures is essential for improving patient quality of life [7]. In 2011, Rossi et al. reported on one of the first large cohorts of patients with skeletal metastases treated with selective arterial embolization. In their study, significant pain palliation and tumor size reduction was achieved using lipiodol/N-butyl cyanoacrylate as embolic materials, slowing disease progression [4]. Nowadays, embolization for bone metastases has gained recognition in the multidisciplinary tumor board for managing highly vascular tumors, especially in cases where radiotherapy is ineffective and traditional surgical approaches are deemed unfeasible or not indicated [8].

Embolization can be performed before surgery to reduce blood loss and complications. Its usefulness has been mostly demonstrated in spinal metastases, where minimizing blood loss during surgery is crucial for reducing complications and improving outcomes [9]. A recent systematic review and meta-analysis demonstrated that embolization significantly decreases intraoperative blood loss in patients undergoing decompressive spinal surgery for metastases [10]. This finding is supported by a growing body of literature showing the benefits of embolization as both a preoperative and palliative treatment.

More recently, newer applications of embolization have been found in the field of musculoskeletal oncology. One example is the treatment of benign bone and soft tissue tumors. Aneurysmal bone cysts (ABCs) are benign, though locally aggressive, and can lead to pathological fractures and spinal cord damage since they often arise in the spine [11]. Cevolani et al. compared the efficacy of embolization with curettage and bone grafting in a large cohort of patients, finding that embolization offered comparable healing rates with fewer complications, particularly in cases where surgery was challenging due to the tumor’s location [12]. Some soft tissue tumors such as Desmoid tumors, a rare type of soft tissue tumor characterized by aggressive local growth but no metastatic potential, could benefit from embolic treatment [13]. Surgical resection has demonstrated to be ineffective due to the tumors’ tendency to infiltrate surrounding structures [13]. Recent studies have proposed the use of arterial embolization with a chemotherapeutic agent as an embolic material; for example, transarterial chemoembolization (TACE) with doxorubicin-eluting beads is a promising alternative for extra-abdominal desmoid tumors, showing significant reductions in tumor volume and symptom relief [14].

In the last decade, arterial embolization has also gained a lot of attention for its role in treating degenerative musculoskeletal conditions. Knee osteoarthritis, one of the major causes of disability in older patients, is typically treated with infiltrative therapies with scarce results or with total knee arthroplasty, which is often poorly tolerated, especially in early onset osteoarthritis. In 2015, Okuno et al. first reported significant pain relief and functional improvement in patients with mild to moderate osteoarthritis after undergoing transcatheter arterial embolization [5]. After his experience, a large number of studies followed, including some prospective studies [15]. Soon, the results of randomized controlled studies comparing arterial embolization with sham techniques will be available [16]. Other chronic pain conditions have been treated following the same principles, most notably tendinopathies and overuse sports injuries, in which embolization has shown promising results in reducing pain and improving function [17].

The goal of this review is to provide an in-depth analysis of the literature on the use of arterial embolization across a variety of musculoskeletal conditions, starting from oncology to degenerative disease. By summarizing the key findings from the most recent studies, this review will assess the efficacy, complications, and recurrence rates associated with embolization. The goal is to provide a comprehensive understanding of how arterial embolization is transforming the treatment of musculoskeletal diseases and to highlight potential areas for future research and clinical innovation.

## 2. Oncologic Embolization

### 2.1. Bone Metastases

Metastatic lesions often exhibit varying degrees of vascularity, with some being highly vascular due to the development of abnormal blood vessels [18,19]. Hypervascular metastases are particularly difficult to treat due to the risk of significant bleeding during surgery. Selective arterial embolization has emerged as a valuable palliative tool in managing these lesions by reducing tumor vascularity and providing substantial pain relief [7,20]. Pain, functional limitations, and an increased risk of pathological fractures are only a few of the ways that bone metastases significantly impair patients’ quality of life [21,22]. Radiotherapy is the gold standard treatment for painful bone metastases, despite a number of side effects, including some tumors’ radio-resistance and treatment-related variability, which is rarely complete [23].

As a result, since the 1970s, arterial embolization has taken on a more significant role in the treatment of metastatic bone lesions, serving as a reliable and safe substitute for patients who cannot undergo surgery [24,25].

**Figure 1:**
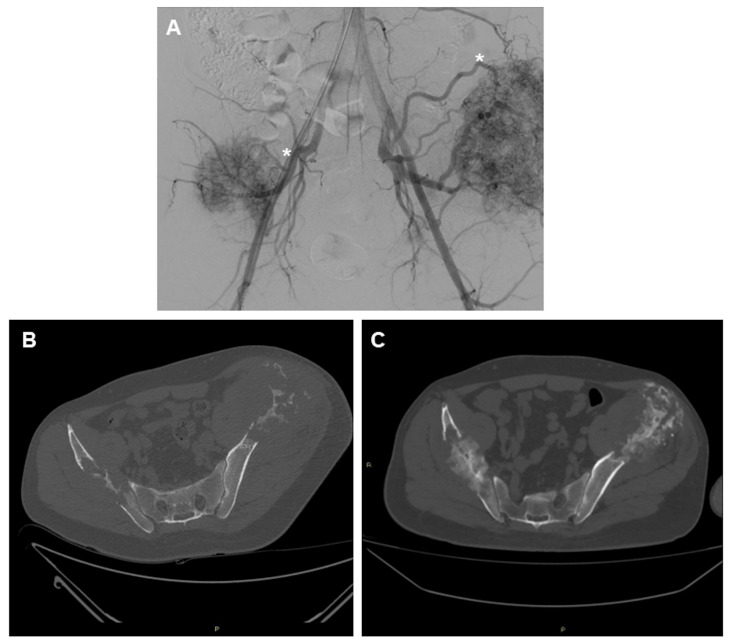
A 56-year-old male patient with two large metastases from renal cancer localized in both iliac bones. (**A**) panoramic DSA angiogram showing two large hypervascular masses supplied by hypertrophic vessels arising mainly from in internal iliac artery (asterisk). (**B**) Pre-embolization CT-scan demonstrating the two large lytic lesions of both iliac bones and (**C**) 6-month follow-up CT showing marked calcification of the lesions.

Unquestionably, transarterial embolization offers the benefit of combining palliative care with the idea of local tumor control by blocking the terminal tumor’s feeding vessels, which causes the tumor to devascularize and shrink (Figure 1). The main studies reporting on the embolization of bone metastases are summarized in Table 1.

### 2.2. Preoperative Embolization

Preoperative arterial embolization is a crucial factor in decreasing intraoperative blood loss, enhancing tumor visualization, and reducing the duration of surgery for patients who are eligible for it [10,26,27,28,29,30,32,35,36,37,38,39,42,44,45]. In a study by Kato et al., intraoperative blood loss was compared between complete and incomplete devascularization in 58 patients with bone metastases from thyroid and renal cancer who had undergone preoperative embolization. Complete embolization resulted in less intraoperative blood loss than partial embolization (mean ± standard deviation, 809 ± 835 vs. 1210 ± 904 mL, *p* = 0.03). Patients who underwent complete embolization also experienced less intraoperative blood loss if their surgery was done on the same day as the embolization [33].

Preoperative embolization was shown to be more successful in minimizing blood loss when surgery was scheduled for the same day as the embolization, according to a recent study that included 41 patients with metastases of renal cell carcinoma in the spinal and extra-spinal regions [40]. In a randomized controlled trial, Clausen et al. included 45 patients with spine metastases from various primary malignancies; 23 of them underwent preoperative embolization, whereas the remaining 22 underwent surgery without preoperative embolization. Preoperative embolization resulted in shorter operating times for the patients, although a statistically meaningful decrease in blood loss was only observed in those with hypervascular metastases [36].

To further emphasize the fact that the choice of the type of tumor is crucial, a recent study [43] evaluated 495 patients with spinal metastases from non-hypervascular primary tumors, 54 of whom underwent preoperative embolization (PE). After propensity score matching, no significant difference in intraoperative blood loss was observed between the embolization group (median 0.9 L) and the non-embolization group (median 0.6 L; *p* = 0.32). Additionally, no significant differences were found in terms of transfusion requirements, anesthesia time, hospitalization duration, postoperative complications, or survival rates, concluding that preoperative embolization may not benefit patients with non-hypervascular spinal metastases.

Less information is available for metastases to bones outside of the spine. However, the majority of data that support the significance of preoperative embolization concerns spinal metastases, with a recent meta-analysis [10] demonstrating that preoperative embolization for spinal metastases was associated with a significant reduction in intraoperative blood loss, with a mean decrease of 1226.9 mL (*p* = 0.006) after excluding the results of one study. However, some authors suggest that preoperative arterial embolization may increase the risk of wound complications in large hypervascular pelvic bone malignancies, which often require extensive embolization [46].

A recent meta-analysis included seven studies reporting the results of preoperative embolization in metastases localized in long bones in terms of blood loss and blood replacement reduction. The level of evidence supporting the effectiveness of preoperative embolization in terms of blood loss and transfusion requirements was low, probably due to the retrospective nature of all studies and the small sample of patients, the lack of standardization of the embolization procedures, and the heterogeneity of the primary tumor type [47].

### 2.3. Palliative Embolization

Many studies have investigated the palliative potential of arterial embolization [20,31]. One of the first large cohort studies was conducted by Rossi et al., with a retrospective analysis of 309 embolizations in performed in 243 patients, with a pain reduction greater than 50% in 97% of procedures [4], and a mean duration of pain relief of 8.1 months. Although pain relief was temporary, a second embolization was performed in order to sustain the pain control. Variable levels of ossification were also noted in 65 patients. Minor complications were observed in 28% of cases, the most common being post-embolization pain, local paresthesia, and skin breakdown.

In a subsequent study including 107 patients with bone metastases from renal cell carcinoma, embolization using N-butyl cyanoacrylate (NBCA) achieved similar results, with pain reduction greater than 50% observed in 96% of cases and an average pain relief duration of 10 months [34]. Tumor size was also significantly reduced, with a mean decrease from 8.8 cm to 4.0 cm following embolization [34].

### 2.4. Combination Therapies

Heianna et al.’s [48] comparative study, which involved 25 patients with bone metastases from renal cell carcinoma, showed that transarterial embolization of a bone lesion can be successfully combined with the administration of a chemotherapy drug in chemoembolization (TACE) [49], ultimately combined with radiation therapy (RT). As compared to radiation alone, the results showed that radiotherapy with TACE had superior radiological objective response and post-RT skeletal-related event-free rates [49].

The effectiveness and safety of transarterial chemoembolization for the palliation of symptomatic bone metastases refractory to first-line radiation have also recently been compared with patients undergoing reradiation by Heianna et al. [50]. Fifty patients were included in the trial, which found that transarterial chemoembolization resulted in palliation that lasted longer in comparison with radiotherapy, with a more substantial tumor size reduction in the TACE group [50].

A study by Zhang et al. [51] compared a cohort of 50 patients receiving percutaneous osteoplasty (POP) with 50 patients which received a combination of POP and transcatheter arterial chemoembolization (POPTACE) for pelvic bone metastases. Both patient groups showed significant improvements in pain and functional recovery. However, in the POPTACE group 74% of patients achieved partial response at 1 month compared to 52% in the POP group (*p* = 0.04). Both groups had similar rates of complications, with asymptomatic cement leakage occurring in 10% of the POP group and 8% in the POPTACE group.

### 2.5. Challenging Locations

Focusing on spinal metastases alone, Facchini et al. [8] performed a retrospective analysis on a group of 164 patients who had 178 selective arterial embolization. A total of 97% of the cases (159 out of 164) had a clinically successful outcome in terms of a decrease in pain score and the requirement for analgesic medication. The efficacy of the treatment lasted an average of 9.2 months (out of a range of 1–12) [8]. When performing spinal embolization, particular attention must be paid to specific side effects of this delicate location. In a systematic review [52] on complications in spinal embolization, the most common injury mechanism was spinal cord ischemia, which occurred in about 1% of cases due to the non-target embolization of arteries supplying the spinal cord. Additionally, cranial infarctions, accounting for 0.6% of cases, were linked to unintentional embolic migration through vertebral or carotid arteries [52].

Embolization has also proven effective for metastases in less common sites, such as the sternum [41], achieving pain reduction greater than 50% after the procedure in all cases. The average duration of pain relief was 9.5 months, and embolization was repeated in four patients in case of pain relapse. No embolization-related complications were reported [41].

By occluding the feeding vessels of the bones metastases, embolization is an effective tool to alleviate pain and reduce mechanical instability caused by tumor growth, improving the quality of life in patients who may not be candidates for surgery or whose metastases did not respond to radiation or chemotherapy. There are limited data on the long-term duration of pain control; however, palliative embolization remains a valuable option for the local control of metastases.

#### 2.5.1. Hemangioma

Vertebral hemangiomas are the most common primary spinal bone lesion, affecting approximately 10–12% of the population [53,54]. Hemangiomas are typically asymptomatic incidental findings and do not require treatment [55]. If they extend beyond the vertebral body to the spinal canal or neural foramina, they are considered locally aggressive or extensive, also called “aggressive hemangioma” [56], eventually becoming symptomatic and causing back pain and neurologic deficits [55,57]. Treatment options for symptomatic or locally aggressive lesions include radiotherapy, isolated decompressive surgery, vertebroplasty, arterial embolization, or a combination of these therapies [48,58].

When surgery is indicated in cases of aggressive hemangioma, preoperative embolization is a valuable adjuvant treatment that reduces intraoperative blood loss and associated morbidity caused by surgery, frequently resulting in massive intraoperative bleeding due to the high vascularization of the lesions [59,60].

In this regard, Teferi et al. [61] reported their experience on the surgical management of aggressive hemangioma, which includes preoperative embolization. In their series, there was a significant reduction in median intraoperative blood loss from 600 mL in non-embolized cases to 395 mL in embolized cases. They also reported improved surgical field visibility and reduced complications, concluding that preoperative embolization improves the surgical management of aggressive vertebral hemangiomas [61]. Arterial embolization could aid other minimally invasive procedures for vertebral hemangiomas, as CT-guided alcohol injection is safer when spinal angiography and arterial embolization of the blood supply to the vertebral body are performed before the lesion is directly transpedicularly punctured [62].

The literature on the embolization of soft tissue hemangiomas is limited compared to that on bone hemangiomas. Embolization may be useful in high-flow soft tissue hemangiomas, where it can help manage symptoms and reduce lesion size by decreasing blood flow to the tumor [63].

A study by Mavrogenis et al. [64] retrospectively reviewed 31 patients treated for painful bone and soft tissue hemangiomas with embolization. For soft tissue hemangiomas specifically, the procedure provided complete pain relief in 10 out of 16 patients, with recurrences in a subset that were successfully managed with repeat embolizations.

#### 2.5.2. Aneurysmal Bone Cysts

Aneurysmal bone cysts (ABCs) are benign, osteolytic bone lesions, characterized by a hypervascular cystic bulge divided by fibrous septa growing within the bone in an expanding intraosseous osteolytic lesion [11,65]. Although ABCs are mostly benign, they are categorized as intermediately malignant tumors since they have the potential to be locally aggressive and cause cortical damage [66,67].

**Figure 2:**
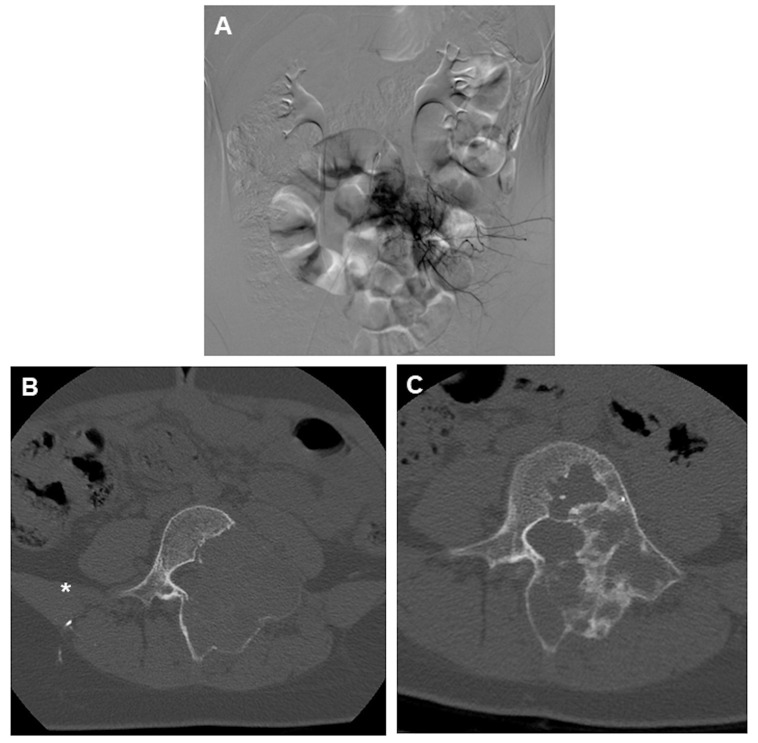
(**A**) DSA angiogram of an ABC of L4 showing marked hypervascularity of the lesion and (**B**) the classic appearance of an aneurysmal bone cyst of the spine, with a lytic lesion surrounded by expanded, thin cortical bone. CT was performed right after the embolization, showing the presence of embolic material (asterisk). (**C**) 1 year follow-up post embolization showing marked recalcification of the lytic lesion.

Selective arterial embolization has a dual role in the management of ABCs: it can be used as a standalone strategy that doesn’t involve additional treatments (Figure 2) or as a preoperative treatment adjuvant to surgery [68]. It is an ideal option for challenging anatomical sites or potentially dangerous surgical procedures [69,70].

Moreover, embolization has the potential benefit of being a less invasive, less expensive, and simpler procedure compared to surgery, making it easily reproducible in the event of recurrence [11,66].

### 2.6. Treatment Through Embolization

The role of arterial embolization in the treatment of aneurysmal bone cysts has been investigated by several studies. Spinal and pelvic aneurysmal bone cysts have shown to benefit more from embolization. A recent study by Cevolani et al. [12] comparing the efficacy of embolization with the traditional approach of curettage and bone grafting included a cohort of 265 patients. Forty-six patients were treated with embolization, with a 58.7% ossification rate after the first embolization, and an overall healing rate of 82% after repeated embolization. The curettage and bone grafting group had a 75.3% success rate but with a slightly higher recurrence rate of 24.7%. In their study, embolization showed a slightly lower complication rate (6%) compared to surgery [12].

In a larger study involving 102 patients, Rossi et al. [71] reported a 81.8% complete response rate after embolization using N-2-butyl cyanoacrylate; the median follow up was 7 years. Recurrence was observed in 18.2% of cases, particularly in younger patients and in lesions larger than 6 cm. The study also reported a low complication rate of 4.5%, including skin necrosis and nerve-related issues [71].

### 2.7. Combination Therapy

For refractory and non-resectable ABCs, combination therapy using selective arterial embolization and percutaneous sclerotherapy has been shown to be a highly effective approach. Masthoff et al. [72] investigated the combination of selective arterial embolization and sclerotherapy, including a cohort of 16 patients, achieving complete or partial response in all cases with no recurrences. Bone mineralization was significantly increased with a reduction in the presence of fluid-fluid levels, along with a reduction in lesion volume. Quality of life change was measured using Musculoskeletal Tumor Society (MSTS) scores, reporting an increase from 14.1 to 28.8 after treatment (*p* < 0.0001) [72].

Combination with curettage is also considered an effective treatment, especially for pediatric cases; a recent meta-analysis [73] analyzing 3467 aneurysmal bone cysts resulted in arterial embolization being a highly effective treatment, especially when combined with curettage, with a recurrence rate of 10%. The complication rate for embolization was low, at 12%.

### 2.8. Preoperative Embolization

As most of the available literature on preoperative embolization of hypervascular tumor suggests, a recent study [66] compared the results of 10 aneurysmal bone cyst patients who underwent preoperative embolization with 9 who did not. The results showed that preoperative embolization significantly reduced intraoperative blood loss (550 mL vs. 1250 mL, *p* = 0.011) and blood transfusion requirements (270 mL vs. 800 mL, *p* = 0.017). No major complications related to embolization were reported.

#### 2.8.1. Giant Cell Tumors

Giant cell tumors (GCTs) of the bone are locally aggressive, benign tumors with a high risk of recurrence after surgical resection; therefore, their management is extremely complex [74]. Although their typical location, the distal epiphysis of long bones, is often treated surgically with good results, some less common locations like the sacrum or pelvis could pose a significant challenge to the standard surgical technique due to their vascular nature and proximity to neurovascular structure [75,76]. Denosumab, a monoclonal antibody targeting RANKL, has become an important tool in managing unresectable GCTs or those with significant risks of morbidity from surgery. Studies including the review by Nagano et al. (2022) [77] have demonstrated its efficacy in reducing tumor size and facilitating less morbid surgeries. However, concerns remain regarding its association with an increased local recurrence rate when used preoperatively. This risk is attributed to factors such as irregular ossification and difficulty in achieving clean margins during curettage [78]. As a result, several studies have reported on the use of selective arterial embolization for the treatment of these challenging locations, either as a standalone therapy or as an adjuvant to surgery, with good long-term results [74,79,80].

A systematic review [81] evaluated the outcomes of arterial embolization in patients with unresectable sacral or pelvic GCTs. During a mean follow-up of 85.8 months, the radiologic response rate was 81.8%, with a local control rate of 75%. None of the patients experienced bowel, bladder, or sexual dysfunction. The 2-, 5-, and 10-year overall survival rates were 90.9%, 88.6%, and 81.8%, respectively. For unresectable cases, arterial embolization is a valid technique capable of providing a good local control of the disease, with minimal side effects.

#### 2.8.2. Osteosarcoma, Ewing’s Sarcoma, and Chondrosarcoma

The most frequent primary malignant bone tumor in children and adolescents is osteosarcoma [82]. Approximately two to three cases per million people are diagnosed with osteosarcoma each year, accounting for 0.2% of all malignant tumors [83].

Mavrogenis et al. reported the biggest cohort of patients with metastatic or unresectable osteosarcoma who underwent palliative embolization. The location was mostly pelvis and lower spine, and the embolic material was N-2-butyl cyanoacrylate. Significant pain reduction and tumor necrosis was observed. No major complications were noted. The study concluded that embolization effectively provided pain relief, but its role in survival improvement is limited [84].

Chu et al. treated 32 patients with osteosarcoma with transarterial chemoembolization prior to limb-salvage surgery [85]. After examining the sample, 85.5% of the patients exhibited significant tumor necrosis following TACE, facilitating easier tumor resection, and it appeared that adding chemotherapeutic agents to the embolization improved the outcome [81]. A high necrosis rate might be crucial for osteosarcoma, where achieving clean surgical margins is essential for long-term control.

Similar results were reported by Zhang et al. [86], in which 47 osteosarcoma patients were treated with intra-arterial chemotherapy combined with embolization prior to limb-salvage surgery, achieving an average tumor necrosis rate of 82.9%. They also compared intraoperative blood loss with a similar control group, and reported that blood loss was significantly lower in the embolized group (290 mL vs. 430 mL, *p* < 0.05) [86].

Limited evidence is available for Ewing’s sarcoma or chondrosarcoma, with most of the studies reporting on the efficacy of preoperative embolization for various musculoskeletal tumors, mostly metastases [87,88,89].

#### 2.8.3. Desmoid Fibromatosis

The term “desmoid fibromatosis” (DF), also known as “desmoid tumor” or “aggressive fibromatosis”, refers to a rare and locally aggressive monoclonal fibroblastic growth that has a fluctuating and frequently unpredictable clinical history [90,91]. Less than 3% of soft tissue neoplasms are identified as DF; their incidence is estimated to be between 3 and 5 cases per million, peaking between the ages of 30 and 40, and being more common in women. The majority of DF are sporadic and are found extra-abdominally in the trunk or extremities [92,93,94]. Since the natural course of DF is unpredictable due to the possibility that tumors will either grow or, conversely, stay stationary or even remiss partially or entirely, new guidelines recommend that the first approach for DF should be watchful waiting [86,91,95].

**Figure 3:**
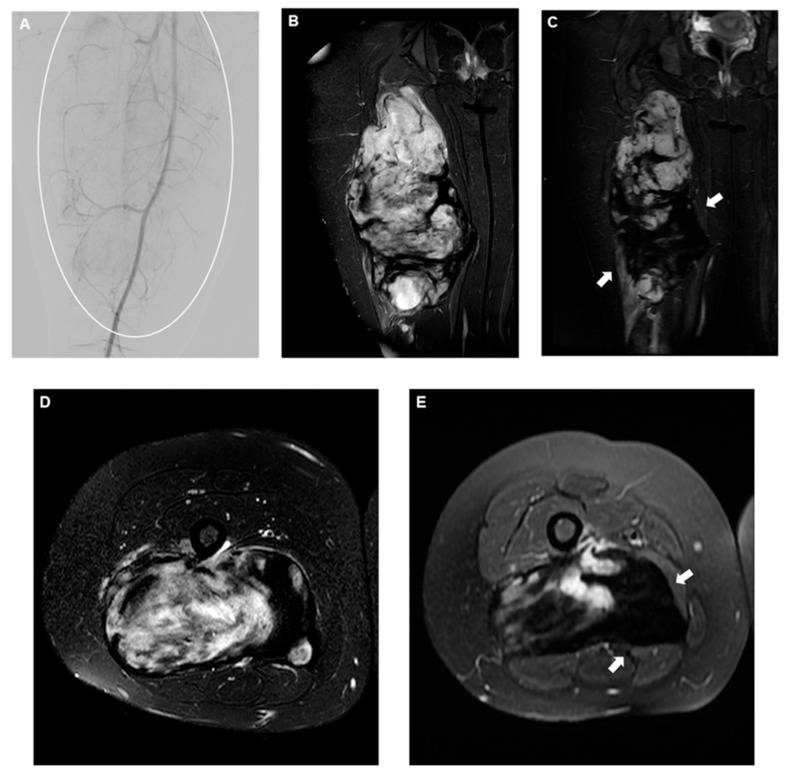
(**A**) DSA of the superficial femoral artery showing a slightly vascularized large mass encompassing the majority of the leg (circle), and (**B**) coronal and (**D**) axial T2-fat saturated pre-procedural MRI demonstrating the classic appearance of a very active desmoid fibromatosis, mostly characterized by high-intensity T2 signal areas. Four-month follow-up coronal (**C**) and axial (**E**) T2-fat saturated MRI showing a significant decrease in T2 signal intensity (arrows), corresponding to inactive disease following embolization. Embolic material used was 300–500 microns microspheres.

In case of documented growth or symptomatic tumors, intervention is required (Figure 3), and current protocols suggest selecting between systemic treatments, primarily consisting of tyrosine kinase inhibitors and chemotherapy [91,96], including Pazopanib [97] and local therapies, with percutaneous cryoablation being one of the most recent and successful available options [98,99,100].

When local treatment is deemed appropriate, however, cryoablation is not always feasible, since in some cases the tumor has an irregular shape due to prior surgery, or is infiltrating neurovascular structures potentially limiting aggressive ablation margins with an increased risk of recurrence [101,102,103].

To address this problem, arterial embolization using doxorubicin drug-eluting (DEE) beads was first proposed by Elnekave et al. [104]. This followed the rationale that systemic doxorubicin is effective in the treatment of DF [105,106], and therefore, embolization could maximize doxorubicin efficacy locally while minimizing systemic toxicity. In their first experience they treated four children with recurrent or refractory desmoid tumors using this approach. Over a follow-up of 6 to 32 months, tumor volumes were reduced by 54% to 97%, with minimal complications, indicating the potential of DEE as a promising treatment option [104].

Following his experience, Kim et al. [5] treated 11 female patients with extra-abdominal DF using the same DEE bead chemoembolization, resulting in significant tumor necrosis, with an average tumor volume reduction of 38.1%.

The most recent study by Elnekave et al. expanded on earlier findings by investigating the long-term outcomes of DEE in 24 patients with DF. With a median follow-up of 8 months, tumor volumes decreased by 59%, with 39% of patients achieving a partial response and 52% experiencing stable disease. The treatment was well tolerated, with only one reported adverse event (grade 3–4).

To minimize non-target embolization, Páez-Carpio et al. [107] reported on the successful prevention of cutaneous complications during DEB-TACE through the administration of subcutaneous epinephrine. This technique allowed for safe and effective tumor devascularization while avoiding skin necrosis.

#### 2.8.4. Degenerative

Since arterial embolization has proven effective in other domains—mostly oncology and trauma—the idea of using it for degenerative joint disease is relatively new. Chronic inflammation is a feature of many musculoskeletal disorders, including osteoarthritis, that results in angiogenesis leading to abnormal hypervascularity [108,109]. According to studies, neuropeptides released by these aberrant arteries may cause nerve growth and persistent pain [110]. Targeting the hypervascular sites, embolization aims to break the cycle of inflammation and stop the growth of sensory nerves, ultimately resulting in alleviating joint pain and improving functional mobility [111].

This idea has been first successfully used to alleviate knee pain related to knee osteoarthritis (KOA): genicular artery embolization (GAE) was found to dramatically reduce pain and boost function in patients with mild to moderate OA [7,112]. Following the success of GAE with OA, musculoskeletal embolization has been applied to other inflammatory conditions such as adhesive capsulitis (AC), lateral epicondylitis (LE), and other isolated localizations of tendinitis [17,112]. In recent years, a great body of literature has been published on the topic, including some randomized controlled trials. Therefore, in this chapter we will try to summarize the main results of the leading studies on GAE and other inflammatory conditions, focusing on different techniques, pain reduction, and complications. Studies reporting on embolization for knee osteoarthritis are summarized in Table 2, and studies reporting on embolization for other locations are summarized in Table 3.

### 2.9. Knee Osteoarthritis

Knee osteoarthritis (KOA) is a disabling condition with high prevalence in the older population that significantly impacts the quality of life for millions of people globally [113,114,115]. Although total knee arthroplasty has long been considered the definitive treatment for advanced cases, many patients with mild to moderate OA remain ineligible for surgery, yet continue to suffer from chronic knee pain and functional limitations [116,117,118,119]. In the last decade, genicular artery embolization (GAE) has proven to be a viable alternative for the large share of patients that are not candidate for knee replacement or who refuse surgery [120]. As stated above, the concept behind GAE is the temporary or permanent occlusion of the abnormal neovascularization caused by chronic inflammation within the knee joint [121]. This chapter will summarize the path that led to the establishment of GAE as a valid therapeutic option, reviewing the most relevant studies on the topic and discussing the various techniques and peculiarities.

In 2015, Okuno et al. [7] treated 14 patients with mild to moderate OA using imipenem/cilastatin sodium or 75 μm Embozene microspheres as the embolic agents, with WOMAC pain scores improving from 12.2 ± 1.9 at baseline to 1.7 ± 2.2 at four months.

In 2017, Okuno et al. [122] expanded their cohort to 72 patients and provided midterm follow-up data. At 24 months, the study reported that WOMAC pain scores decreased from 12.1 to 2.6, with an 86.3% clinical success rate at six months and 79.8% at three years. Their results paved the way for a new research path that would soon be followed by many IR teams around the globe [123,124,125,126,127,128,129,130,131,132,133,134] (Table 2).

**Table 2:** Main articles covering the topic of arterial embolization for knee osteoarthritis. (KL: Kellgren–Lawrence).

N	First Author, Year	Reference N	Study Design	N of Patients	Mean Age	KL Score	Previous Treatments	Median Follow-Up in Months	Embolic Material Used	Complications	Results
1	Okuno, 2015	[7]	Prospective	14	65.2	0, 1, 2	3 months of conservative therapies (anti-inflammatory drugs, PT, muscle strengthening, and intra-articular injection of hyaluronic acid)	12	IPM/CS, 75 um calibrated Embozene microspheres	1 moderate subcutaneous hemorrhage	The mean WOMAC pain score of all treated patients significantly decreased from 12.2 to 3.3 at 1 month after the procedure, with further improvement at 4 months (1.7), and the mean WOMAC total score decreased from 47.3 to 11.6 at 1 month, and to 6.3 at 4 months. These improvements were maintained in most cases at the final follow-up. The mean overall VAS scores before treatment significantly decreased at 1 week and at 1 and 4 months thereafter (70 vs. 29, 21,and 13). The dose of medication and the frequency of injection therapy decreased after procedure.
2	Okuno, 2017	[122]	Prospective	72	64.4	1, 2, 3	3 months of conservative therapies (NSAIDs, oral opioids, PT, stretching, muscle strengthening, or intra-articular injection of hyaluronic acid)	24? NA	IPM/CS, 75 um calibrated Embozene microspheres	12 moderate subcutaneous hemorrhage, 4 transient cutaneous skin changes	Mean WOMAC scores significantly decreased from baseline to 1, 4, 6, 12, and 24 months after treatment (12.1 vs. 6.2, 4.4, 3.7, 3.0, and 2.6; all *p* < 0.001). The cumulative clinical success rates at 6 months and 3 years after embolization were 86.3% and 79.8%, respectively. WORMS scores at 2 years after embolization in 35 knees showed significant improvement of synovitis vs. baseline.
3	Lee, 2019	[135]	Retrospective	41	66.2	1, 2, 3, 4	3 months of conservative treatments (PT, muscle strengthening, NSAIDs, intra-articular hyaluronic acid injection therapy)	10	IPM/CS	5 subcutaneous haematomas at the puncture sites, 4 skin redness, 1 mild fever	Transcatheter arterial embolization effectively relieved pain in patients with mild to moderate osteoarthritis. In patients with severe osteoarthritis, pain severity decreased for 1 month but gradually increased to the initial severity score within 3 months.
4	Little, 2021	[136]	Prospective (interim analysis)	38	60	1, 2, 3	6 months of conservative treatments	8	100–300 μm permanent micro-spheres	4 skin discoloration, 1 hematoma	Mean VAS at baseline was 60, reducing to 32 at 6 weeks, 36 at 3 months, and 45 at 12 months. KOOS subscales showed a statistically significant improvement from baseline to 6 weeks, 3 months, and 1 year in all outcome measures except function in daily living, which revealed borderline significance at 12 months.
5	Landers, 2020	[137]	Prospective	10	62.2	1, 2	6 months of failed conservative treatment	21.6	Polyvinyl embolic material, IPM/CS	1 hematoma	Six patients (60%) demonstrated a response to treatment at the 12-month assessment. Seven patients (70%) were responders at 1 month and 6 months, and 3 patients (30%) were responders at 24 months. Of the 3 responders at 24 months, 2 had received repeat embolization after the 12-month assessment.
6	Bagla, 2020	[138]	Prospective	20	59.4	1, 2, 3	3 months of conservative therapy (pain medication or intra-articular injections)	6	75 μm Embozene, 100-μm particles	13 skin discoloration, 2 plantar paresthesia	Embolization of at least one genicular artery was achieved in 20/20 (100%) patients. Mean VAS improved from 76 mm ± 14 at baseline to 29 mm ± 27 at 6-month follow-up (*p* < 0.01). Mean WOMAC score improved from 61 ± 12 at baseline to 29 ± 27 at 6-month follow-up (*p* < 0.01).
7	Padia, 2021	[123]	Prospective	40	69	2, 3, 4	3 months of conservative treatment (NSAIDs, PT, and joint injection)	12	100 μm Embozene particles	1 groin hematoma, 7 focal skin necrosis resolved with ice packing, transient skin discoloration, 3 bone infarction	Twenty-seven (68%) of the 40 subjects achieved clinical success from GAE, defined as a reduction of at least 50% in the WOMAC total score from baseline to 12 months. Furthermore, 17 (43%) of the 40 subjects had a reduction of >75% in the WOMAC score at 12 months. Twenty-seven (68%) of the forty subjects reported a reduction in pain on the VAS of >50% from baseline to 12 months.
8	van Zadelhoff, 2021	[139]	Retrospective	54	69.4	1, 2, 3, 4	3 months of conservative therapy	6	IPM/CS	NA	Six months after GAE, the median WOMAC pain reduction was 8 points, and the mean WOMAC total reduction was 24.2. Of all analyzed features, the cartilage full-thickness score showed the strongest association with a reduction of both the WOMAC pain and the WOMAC total score.
9	Bagla, 2022	[124]	RCT	21	62.9	1, 2, 3	3 months of conservative therapies (medication, PT, or intra-articular injections)	12	100–300 μm OptiSphere absorbable particles	Knee pain, purpura, nausea/vomiting, hematoma, skin changes, skin ischemia, pruritus, ecchymosis, bleeding at access site, 3 patients drop out due to increased pain	All subjects in the sham group failed to show significant improvements at 1 month and crossed over to the treatment arm. There was a statistically significantly greater pain reduction in the treatment group than in the sham group at 1 month (VAS, 50.1 mm; standard error [SE], 10.6; 95% confidence interval [CI], 29.0, 72.3; *p* < 0.01). Disability improvement was also significantly greater in the treatment group (WOMAC, 24.7 points; SE, 10.4; 95% CI, 3.5, 45.9; *p* = 0.02).
10	Bhatia, 2023	[125]	Retrospective comparative	21	73.14	2, 3, 4	6 months of conservative management (PT, NSAIDs, or intra-articular injection of hyaluronic acid or steroids)	26.1	100–300 μm trisacryl gelatin microspheres, IPM/CS	ES group: 3 transient cutaneous color change, 1 transient leg numbness, NO AE in IMP/CS group	There were no significant differences in clinical outcome measures at the 3-month or 24-month follow-up. Both embolic materials resulted in a decrease in WOMAC pain and WOMAC total scores at 3 months (*p* < 0.05), and the effect of treatment on WOMAC pain and WOMAC total score reduction was sustained until the 24-month follow-up (*p* < 0.05).
11	Taslakian, 2023	[126]	Prospective (interim analysis)	27	67.1	2, 3, 4	3 months of conservative therapy	6	250-μm Embozene microspheres	1 hematoma	The mean WOMAC pain score decreased from 8.6 ± 2.7 at baseline to 4.9 ± 2.7 (*p* = 0.001), 4.4 ± 2.8 (*p* < 0.001), and 4.7 ± 2.7 (*p* = 0.094) at 1, 3, and 12 months, respectively. There was a statistically significant decrease in nerve growth factor (NGF) levels at 12 months.
12	Wang, 2023	[127]	Prospective	22	63.5	1, 2, 3	>3 months of conservative treatment (anti-inflammatory drugs, PT, muscle strengthening, or intra-articular injections)	5.7	IPM/CS	4 moderate subcutaneous hemorrhage at the puncture site, 3 transient cutaneous color change	GAE significantly decreased the VAS scores at 3 and 6 months after embolization in patients without BML (both *p* = 0.04) and those with BML (both *p* = 0.01). GAE also lowered the WOMAC scores 3 months after embolization in patients without and with BML (*p* = 0.02 and *p* = 0.0002, respectively). However, GAE did not significantly alter the BML area and volume (both *p* = 0.25), VAS scores (*p* = 1.00), and WOMAC scores (*p* = 0.08) in patients with BML and SIFK at 3 months after GAE.
13	Landers, 2023	[128]	RCT	59	60.1	2	6 months of conservative treatment	12	IPM/CS	5 bruising near incision site	Median KOOS scores at 12 months for the complete embolization group (*n* = 17) were significantly better than the control group (*n* = 29) for KOOS Sports and Recreation scale and KOOS Quality of Life scale. For Global Change at 12 months, participants who received complete embolization were better compared to participants in the control group.
14	Min, 2023	[129]	Retrospective	71	64	NA	>6 months of conservative treatment	6	100–300 μm QS-GSPs	49 temporary skin color changes,10 hematoma, 1 mild allergic reaction to iodinated contrast	The mean VAS scores at baseline, immediately after TAE, and at 1 day, 1 week, 1 month, 3 months, and 6 months after TAE were 6.3, 4.0, 5.0,3.0, 2.9, 2.9 and 2.8 respectively. Clinical success was 72.2% at 6 months follow-up.
15	Gill, 2023	[130]	Prospective	33	62.5	2, 3, 4	3 months of conventional therapies (exercise, analgesia, anti-inflammatories, orthotics, and weight loss)	6	IPM/CS	1 cannulation site bruising, 1 skin erythema	Higher proportion of participants (*n* = 9, 81.8%) with mild OA fulfilled responder criteria after treatment compared with people with moderate to severe OA (n = 8, 36.4%) (*p* = 0.014). Secondary outcomes for pain, quality of life, and global change were also better in the mild OA group (*p* < 0.05).
16	Dablan, 2024	[140]	Retrospective	68	59.1	1, 2, 3, 4	Conservative treatments	3	IPM/CS	6 transient skin discoloration	Synovial contrast enhancement scores significantly decreased from 5.1 (SD ± 2.0) to 2.9 (SD ± 2.0) at 3 months (*p* < 0.001), with a moderate negative correlation between synovial enhancement scores and pain levels (*p* = 0.005).
17	Guzelbey, 2024	[131]	Retrospective comparative	79	65	1, 2, 3, 4	Conservative treatments (PT, NSAIDs, intra-articular steroid or hyaluronic acid injection)	6	IPM/CS	5 small hematomas, 23 transient skin discoloration	The technical success rate in the macrocatheter group was determined to be 91%, while it was 100% in the microcatheter group; however, no statistically significant difference was detected between the two groups.
18	Hindsø, 2024	[132]	Prospective	20	56	1, 2, 3	3 months of PT	6	100–300 μm Embosphere^®^ Microspheres	4 transient skin changes and 2 hematoma	The primary endpoint, VAS at six months, showed significant improvement (median reduction from 66 mm to 40 mm, *p* = 0.0004). All pain and function scores, as well asphysical performance tests, improved significantly. No clinically relevant changes in medication use or DEXA parameters were observed after six months.
19	Kılıc ¸kesmez, 2024	[133]	Retrospective	60	64	1, 2, 3, 4	3 months of conservative treatments	6	IPM/CS	Entry site hematoma, skin discoloration, transient paresthesia, vasospasm, dissection, and fever.	No significant differences in VAS, WOMAC pain, and WOMAC total scores were identified between TPA and TFA groups at 1, 3, and 6 months post-procedure.
20	Little, 2024	[15]	Prospective	46	60	1, 2, 3	6 months of conservative treatments	17.3	100–300 μm permanent microspheres	4 skin discoloration, 1 hematoma, 1 popliteal deep vein thrombosis	Mean VAS improved from 58.63 at baselines to 37.7 at 2-years. Whole and subgroup KOOS were significantly improved at each timepoint with associated reductions in analgesia usage. WORMS analysis demonstrated significant reduction in synovitis.
21	Sapoval, 2024	[141]	Prospective, multicenter	22	66	3, 4	3 months of conservative treatments (intra-articular corticosteroid injections, analgesic medication)	3	Emulsion 1:3 (v:v) ioversol 300 mgI/mL and ethiodized oil	1 reversible deterioration in renal function (increase in serum creatinine), 1 edema, 1 erythema	Mean VAS pain score decreased from 74.4 mm at baseline to 37.2 mm at 3 months. WOMAC function score at 3 months decreased to 33.5, representing a mean change from baseline of 23.6.
22	Sun, 2024	[134]	Prospective	33	64.5	2, 3, 4	6 months of conservative treatment (pharmacologic therapy, PT, muscle strengthening, or intra-articular injection)	12	Polyvinyl alcohol particles (150–350 μm)	1 localized skin ulcer, 4 skin ecchymosis, 3 knee stiffness and calf muscle pain, 1 occasional knee clicking	The mean VAS and WOMAC scores in the mild to moderate group significantly decreased (6.6 at baseline vs. 3.0 at 12 months and 49.4 vs. 27.4, respectively, all *p* < 0.001). The mean VAS and WOMAC scores in the severe group significantly decreased at 12 months (7.3 vs. 4.4 and 58.1 vs. 40.6, respectively, all *p* < 0.001).
23	Cusumano, 2024	[119]	Prospective	40	66	2, 3, 4	3 months of conservative treatment (NSAIDS/PT/joint injection)	20.1	100 μm Embozene particles	1 groin hematoma, 7 focal skin ulceration, 2 asymptomatic small bone infarct	A total of 18 of 38 (47.4%) patients demonstrated ≥50% reduction in WOMAC at 24 months. In the subset of patients with initial clinical success at 12 months, 18 of 25 (72.0%) reported sustained clinical success at 24 months.

Firstly, Bagla et al. [138] reproduced Dr Okuno’s technique in the Unites States, treating 20 patients with 75 μm–100 μm Embozene, with a clinical success rate of 80% at six months [138]. Landers et al. [137] reported that six out of ten patients with mild to moderate osteoarthritis who were treated with IPM/CS or 90–180 μm of polyvinyl alcohol particles showed a response to treatment at the 12-month follow-up. Their results were followed by Little et al. [136], who treated 38 patients with mild to moderate osteoarthritis, using 100–300 μm Embospheres with a significant improvement in their visual analogue scale (VAS) and Knee Injury and Osteo-arthritis Outcome Score (KOOS) ratings at the 12-month follow-up.

Many trials supporting the efficacy of KOA embolization have followed.

Torkian et al. [142] conducted a systematic review and meta-analysis that included 11 articles, 225 patients, and 268 treated knees. The most used embolic agents were Embozene, imipenem/cilastatin, resorbable microspheres, and polyvinyl alcohol (PVA). They came to the conclusion that even a week after GAE there was a significant improvement in pain [142]. Taslakian et al. [143] conducted another systematic review and meta-analysis, concluding that patients with higher baseline knee pain responded better to GAE. Another systematic review and meta-analysis was completed shortly after by Epelboym et al. [144], concluding that patients who underwent the procedure had significant improvements up to a twelve-month follow-up.

Finally, during the last year, more authors have published their results, further consolidating the data on the technique. The 2-year follow-up of the GENESIS study by Little et al. [15] reported the outcomes of 46 patients, with an improvement of a mean VAS score from 58.63 to 37.7 (95% CI 27.0–47.5), and KOOS pain improved by 24 points. The technical success rate was 87%, with minor complications [15]. Interestingly, in the LipioJoint-1 trial, Sapoval et al. [141] reported positive outcomes using an ethiodized oil-based emulsion as the embolic material. The mean VAS score was reduced from 74.4 mm to 37.2 mm at three months (*p* < 0.001), and WOMAC function improved from 57.3 to 33.5 (*p* < 0.001) [137].

Regarding pretreatment imaging, Zadelhoff et al. [139] correlated baseline MRI features with clinical outcomes of GAE, reporting that patients with more severe osteoarthritis resulted in less pain reduction following GAE. Similarly, Lee et al. [135] reported that patients with mild to moderate knee osteoarthritis had better outcomes compared to those with severe OA, suggesting that GAE may be more effective in earlier stages of the disease. Post-treatment imaging was investigated by Dablan et al. [140], using contrast-enhanced MRI to evaluate the effects of GAE on synovitis, with a significant reduction in synovial contrast enhancement in the parapatellar and periligamentous regions three months after [140].

### 2.10. Adhesive Capsulitis

Adhesive capsulitis (AC) is a debilitating condition characterized by glenohumeral joint discomfort and limited range of motion [145]. Between 2 and 5% of the general population are thought to be affected, with a slight prevalence among females [146,147], more often occurring between the fifth and seventh decades of life [148]. While the exact etiology of adhesive capsulitis is still unknown, the condition is thought to be a predominantly fibrotic disorder, characterized by low-grade inflammation that promotes fibroblast growth and collagen deposition within the glenohumeral joint capsule [149]. The goal of treating adhesive capsulitis is to decrease pain and increase functional mobility in order to restore the joint’s normal functioning [150,151]. Since adhesive capsulitis frequently resolves on its own, the best course of treatment is determined by the patient’s symptoms and medical history, as there is currently no definitive treatment protocol due to the paucity of accessible data [152,153]. The first line of treatment is conservative, including physical therapy, anti-inflammatory drugs, and corticosteroid injections. When conservative treatments are insufficient, employing more invasive methods may become necessary [154].

Arterial embolization for AC was proposed by Okuno et al. in a pilot study conducted in 2014 using imipenem/cilastatin as embolic materials [151], following the same rationale of targeting the pathological blood vessels that contribute to inflammation and pain. His hypothesis was that in AC, increased vascularity and inflammation around the shoulder joint capsule might cause pain and restricted movement. By embolizing these abnormal vessels, predominantly arising from the coracoid branch and thoracoacromial artery [155], arterial embolization could reduce the inflammation, disrupting the pain cycle, and promoting functional recovery [151]. In a murine frozen shoulder model, arterial embolization led to a significant reduction in the number of abnormal blood vessels and mononuclear inflammatory cells in the treated group compared to controls (*p* = 0.002 and *p* = 0.001, respectively) [156]. Following the positive results of his pilot study, in 2017 Okuno et al. [112] reported the outcomes of 25 patients treated with the same imipenem/cilastatin mixture, with a 67% rapid pain relief within one week after embolization, and 87% improvement within one month [112]. As for genicular artery embolization, his experience was followed by several other trials [157,158,159,160,161]. Bagla et al. [162] treated 20 patients with AC, using 75-μm or 200-μm microspheres, achieving a VAS pain score reduction from 74.2 to 22.1 at six months (*p* < 0.001), and improvement in SANE and ASES scores [162]. In 2022 Okuno et al. [163] published a multicenter study with imipenem/cilastatin arterial embolization performed for AC in 76 patients, achieving reduction in nighttime pain, with NRS scores dropping from 6.4 to 1.6 at six months (*p* < 0.001). Range of motion also improved, with anterior elevation increasing from 97° to 151° (*p* < 0.001), and quality of life scores (EQ-5D) rose from 0.63 to 0.84. Clinical success was achieved in 86% of patients [163].

The most recent data were published in a prospective study by Lanciego et al. [164] in 2024, reporting a decrease in nocturnal pain by 320% (*p* = 0.003), reduced pain during movement by 273% (*p* = 0.001), and improved range of motion with active flexion increasing by 80% and external rotation by 72% (*p* < 0.001) at 6 moths. Only one minor adverse event was reported.

### 2.11. Other Locations

Other inflammatory conditions other than knee osteoarthritis and adhesive capsulitis have garnered significant attention, particularly hip osteoarthritis, lateral and medial epicondylitis, lower back spondyloarthritis, and sport-related injuries and tendonitis (Table 3).

**Table 3:** Main articles covering the topic of arterial embolization for degenerative or inflammatory musculoskeletal conditions other than knee osteoarthritis.

N	First Author, Year	Reference N	Study Design	N of Patients	Mean Age	Site	Pathology	Previous Treatments	Follow-Up Months	Embolic Material Used	Complications	Results
1	Okuno, 2013	[157]	Prospective	7	51.7	Knee, shoulder, foot, ankle, elbow	Tendinopathy	3 months of conservative therapies (rest, NSAIDs, ice, stretching, strengthening, corticosteroid injections, PT, and iontophoresis)	4	IPM/CS	1 moderate subcutaneous hemorrhage	Compared with before treatment, mean VAS scores were significantly decreased at 1 day, 1 week, and 1 and 4 months after treatment (72.7 mm vs. 17.4 mm, 16.0 mm, 13.7 mm, and 9.7 mm, respectively; all *p* < 0.001).
2	Okuno, 2014	[151]	Prospective	7	50.3	Shoulder	Adhesive capsulitis	3 months of conservative treatments	10	IMP/CS	0	The mean nighttime VAS score significantly improved from before embolization to 1 week, 1 month, 3 months, and 6 months after embolization (67 mm vs. 27, 6 mm, 2 mm, and 2 mm, respectively; all *p* < 0.001). Overall VAS and ASES scores increased significantly.
3	Iwamoto, 2017	[165]	Prospective	24	52.1	Elbow	Lateral epicondilytis	Conservative therapy: NSAIDs 13, physical therapy 24, steroid injections 22	22.1	IPM/CS or Embosphere 100–300 um	2 transient radial artery spasm	The mean QuickDASH score before TAE significantly decreased at every follow-up visit (50.8 vs. 23.4, 8.3, 5.3, 2.5, and 2.7; all *p* < 0.001). The mean maximum pain VAS score before treatment significantly decreased at 1, 3, 6, 12, and 24 months after the first TAE procedure (77 mm vs. 49 mm,31 mm, 16 mm, 9 mm, and 11 mm, respectively; all *p*< 0.001).
4	Okuno, 2017	[112]	Prospective	25	53.8	Shoulder	Adhesive capsulitis	3 months of conservative treatment (rest, NSAIDs, corticosteroid injections, PT)	36.1	IMP/CS	5 evoked pain, 2 radial artery spasm, 1 puncture site pain, 1 fever	Mean VAS score decreased at 1 week, 1 month, 3 months, and 6 months. At 12 months, 21 of 24 (88%) patients were completely pain free. That rate increased (22 of 24; 94%) at the final follow-up.
5	Hwang, 2018	[158]	Retrospective	13	52.4	Shoulder and elbow	Tendinopathy	6 months pain refractory to conservative treatment	4	Tris-acryl microspheres 40–120 μm, IPM/CS	1 forearm cutaneous erythema	A decrease in the VAS score was noted in 12 of 15 cases (80%). The mean VAS scores at baseline, 1 day, 1 week, 1 month, and 4 months after embolization were 6.1, 5.8, 5.1, 4.3, and 2.5, respectively.
6	Bagla, 2021	[162]	Prospective	20	50.8	Shoulder	Adhesive capsulitis	30 days of conservative therapy (pain medications, PT, injections, etc).	1	75-μm or 200-μm HydroPearl	7 minor skin discolorations, 2 itchiness	The 1, 3, and 6-month follow-ups demonstrated significant improvements according to the VAS, SANE, and ASES scores.
7	Fujiwara, 2021	[166]	Retrospective	14	55.6	Hip	Synovitis	3 months of conservative treatment: NSAIDs, opioids, physical therapy, acupuncture, local steroid injection	20.7	IPM/CS	5 strong evoked pain, 1 puncture site pain, 1 mild subcutaneous hemorrhage	Mean BPI maximum pain intensity scores and ODI decreased significantly at 1, 3, and 24 months after TAE compared to those at baseline.
8	Martinez, 2021	[159]	Retrospective	25	49	Shoulder	Adhesive capsulitis	3 months of PT, NSAIDs, corticosteroid infiltrations	6	IPM/CS	2 groin discomfort	Median pain VAS before TAE was 8. It decreased to 4 at one week, 3 at one month, and 2 at both 3 and 6 months after TAE. At the 6-month follow-up examination, 10 of 25 patients (40%) reported no pain, and only 4 of 25 (16%) reported a pain VAS of more than 7.
9	Martinez, 2021	[160]	Prospective	40	50	Shoulder	Adhesive capsulitis	3 months of PT, corticosteroids infiltrations	12	IPM/CS	2 groin discomfort and hematoma	Before TAE, no patient referred pain VAS < 6 and 28/40 (70%) patients reported pain ≥ 8. Mean pain VAS after TAE decreased to less than 4 in 26/40 (65%) patients at the 1-week follow-up, less or equal to 3 in 28/40 (70%) at the 1-month follow-up and 2.1 ± 1.8 at the 3-month follow-up. At the 12-month follow-up 33/40 (82.5%) reported a progressive decrease in pain (VAS up to ≤3).
10	Correa, 2022	[167]	Prospective	13	62.1	Hip	Osteoarthritis	6 months of conservative management or physical therapy	6	IPM/CS, 100–300 um microspheres embosphere or bead block microsphere for fistulae-like pattern	0	The median WOMAC Index had a statistically significant decrease in the total value from 77 pre-procedure to 27 points after six months (*p* = 0.001). The pain score had a median decrease of 14 points (19 to 5, *p* = 0.001). The rigidity score had a reduction of 6 to 2 points (*p* = 0.002), and the median physical activity score also significantly reduced from 53 to 22 points (*p* = 0.001).
11	Lee, 2022	[168]	Retrospective	10	53.9	Elbow	Medial epicondilytis	Conservative treatments: NSAIDs all, PT 13, ESWT 13	9.8	IPM/CS 12 procedures, quick-soluble gelatin sponge particles 2 pr	3 radial puncture site pain	The mean QuickDASH scores at baseline decreased significantly 1 day, 1 week, and 1, 3, and 6 months after TAE (71.9 versus 48.5, 44, 37.7, 30.2, and 8.4, respectively; all *p* < 0.01). Clinical success 6 months after the procedures was achieved in 12 of 14 cases (85.7%). The mean VAS scores were significantly decreased 1 day, 1 week, and 1 month, 3 months, and 6 months (7.6 at baseline versus corresponding scores of 3.6, 3.6, 3.6, 3, and 0.9 after the treatment; all *p* < 0.01).
12	Okuno, 2022	[163]	Prospective multicenter	100	58.7	Shoulder	Adhesive capsulitis and rotator cuff tear	3 months of PT or steroid injection	5.46	IMP/CS	5 evoked pain, 4 pucture site pain, 2 transient radial artery spasm, 1 fever, 1 difficulty in hearing (possibly unrelated)	A total of 80/93 (86%) demonstrated improvement in nighttime pain by 2 or more in the NRS scores. The mean nighttime NRS scores at baseline and 1, 3, and 6 months after TAE were 6.4 ± 2.2, 3.4 ± 2.6, 2.3 ± 2.5, and 1.6 ± 2.2, respectively (for all, *p* < 0.001).
13	Lanciego, 2023	[164]	Prospective	20	50.7	Shoulder	Adhesive capsulitis	6 weeks of conventional treatment (PT, analgesics, intra-articular infiltration, or suprascapular nerve block)	18	IMP/CS	1 transient edema	Six months after embolization, significant improvements were observed in nocturnal pain, pain on moving, external and internal rotation, active and passive flexion, active and passive abduction, and overall function.
14	Okuno, 2023	[17]	Case series	10	31.3	Knee, foot, wrist, hamstring, lower back	Sports injuries	Conservative treatments: prolonged rest, ice baths, NSAIDs, corticosteroid and PRO injections, ESWT, PT	NA (12 m?)	IPM/CS, Nexsphere-F	NA/0	TAE was effective and provided short-term pain relief. There were cases of gradual improvement with repeated treatment.
15	Shintaku, 2023	[161]	Retrospective	15	54.9	Shoulder	Adhesive capsulitis	NA	2	IPM/CS	NA	The decrease in FDG uptake showed a significant correlation with the change in the pain scale score and extension score. Patients showed improvement in range of motion.

Hip osteoarthritis is a major cause of pain, and arterial embolization could fill the gap between conservative therapies or total hip arthroplasty. This offers pain relief and functional improvement up to 6 months, as reported by Correa et al. [167] on a cohort of 13 patients with hip osteoarthritis and greater trochanteric pain syndrome. The patients received an embolization of the lateral femoral circumflex artery, resulting in a VAS score decrease from a median of 10 to 2 at the 6-month follow-up (*p* = 0.002), and WOMAC index improvement, with total scores dropping from 77 to 27 (*p* = 0.001) [167].

Arterial embolization has proven effective in reducing pain and improving functionality also in lateral and medial epicondylitis, commonly known as tennis or golfer’s elbow respectively, with a significant reduction in VAS scores up to six months and functional improvements observed in the vast majority of patients, with no major complications [165,168].

In a recent study, Okuno et al. [17] also treated 22 athletes with chronic sports injuries, suffering from Achilles tendonitis, patellar tendonitis, and rotator cuff inflammation, amongst others. The study showed a 73% improvement in pain scores, with 81% of athletes returning to full activity within six months, without major complications.

Chronic back pain related to spondyloarthritis and degenerative disc disease has limited treatment options for patients who are not surgical candidates. Arterial embolization was proposed for patients with facet or sacroiliac joint syndrome refractory to conservative treatments in a cohort of 14 patients, with VAS decreasing from 7.5 to 3.0 at six months, without major complications [166].

## 3. Discussion

In modern precision medicine, arterial embolization is an essential treatment for managing a wide range of musculoskeletal pathologies. It has been skillfully refined for over more than 50 years, initially for bleeding control, to preoperative and palliative tumor management in oncologic settings, to many therapeutic indications as a standalone treatment in oncologic and degenerative disease. This review thoroughly compiles and discusses the literature on these topics, summarizing the main findings in tables when a large number of articles are available. Selected case studies are presented throughout the review to enhance the reader’s experience. In the treatment of hypervascular bone metastases, embolization has demonstrated efficacy in pain palliation, local tumor control, and reducing surgical complications. Moreover, in primary bone and soft tissue tumors like aneurysmal bone cysts and desmoid fibromatosis, embolization offers a less invasive alternative to surgery, with promising long-term outcomes and minimal complications. The preferred clinical scenarios for oncologic embolization vary according to the type of lesion (Table 4).

Table 4 summarizes clinical settings and indications for embolization in musculoskeletal pathology.

For bone metastases, large, lytic, hypervascular lesions that do not respond to radiotherapy or chemotherapy are ideal candidates for embolization to achieve pain reduction and local disease control [4,8,9]. For primary bone tumors, the indication is usually related to the location of the lesion, where proximity to neurovascular structures often makes percutaneous or surgical approaches less effective [99,100,101]. It is thought that the decompression of the periosteum is connected to the decrease in pain. Theoretically, embolization decreases the activation of nociceptors by either decreasing the volume of the tumor to a point where endosteal or periosteal tension is released, or by limiting the generation of algesic messengers [169,170]. This theory may help to explain why embolization may result in pain relief [87,171].

In the last decade, following the experience of oncologic embolization, the indications have been extended to degenerative musculoskeletal conditions, with most of the initial studies have focused on knee osteoarthritis and adhesive capsulitis, filling the gap between conservative treatment and surgical intervention.

Limited side effects have been reported in the literature. In oncologic embolization, post-embolization syndrome—defined as fever, nausea and vomiting, and increased ischemic pain—has been observed in roughly 20% of patients, although it is self-limiting within one week after the procedure [4]. For degenerative embolization, side effects appear to be less common and less severe, probably due to the reduced aggressiveness of the embolization itself and the limited ischemic effect. Skin discoloration is the most common side effect, affecting 12.5% of patients [16].

Arterial embolization has certain disadvantages and contraindications. Disadvantages include the risk of non-target embolization leading to unintended tissue damage, radiation exposure during the procedure, and the potential for symptom recurrence necessitating repeat interventions [25,27,28,29,30,32,35,37]. Contraindications encompass patients with severe atherosclerotic disease limiting catheter access, uncorrectable coagulopathy or bleeding disorders, severe renal insufficiency that precludes the use of contrast agents, and hypersensitivity to embolic materials or contrast media [27,28,29,30,32,35,37].

Future perspectives include the development of ever less invasive embolization techniques, made possible by advances in imaging and the development of novel embolic materials. As research in interventional radiology evolves rapidly, long-term data on recurrence and complications are becoming available to physicians worldwide, positioning arterial embolization in the center of the landscape of musculoskeletal disease.

In conclusion, this review covers the vast majority of the current existing applications of arterial embolization in musculoskeletal pathology, from metastatic bone disease to osteoarthritis, with the scope of stimulating further research to provide better patient care, supporting the precise, tailored medicine of the future.

## Figures and Tables

**Table 1:** Main articles covering the topic of arterial embolization for bone metastases. (PrO: Preoperative; Pa: Palliative).

N	Main Author, Year	Reference N	Study Design	PrO/Pa	Primary Tumor	Location of Metastasis	Included	Embolization	Control	Primary Outcome	Complications	Results
1	Chatziioannou, 2000	[26]	retrospective	PrO	Renal cell carcinoma	Femoral/acetabular 17, other 11	26	28	Complete devascularization vs. incomplete devascularization	Blood loss	ND	Complete devascularization reduces blood loss during surgery
2	Guzman, 2005	[27]	retrospective	PrO	Renal 14, 4 thyroid, 6 various	Spine	24	22	Complete devascularization vs. incomplete devascularization; grade of vascularization	Blood loss	ND	Embolization reduces blood loss; no difference between vascularization grade
3	Wirbel, 2005	[28]	retrospective	PrO	Renal 45, other 17	Spine 41, pelvis 21	62	32	Embolization vs. no embolization	Blood loss, blood replacement, operating time	2 m	Embolization reduces blood loss and need for blood replacement
4	Forauer, 2007	[20]	retrospective	Pa	Renal cell carcinoma	Pelvic 18, spine 5, other 16	21	39	0	Pain palliation	1 m, 2 M	Effective pain palliation was achieved in 36/39 sites, average duration 5.5 months
5	Kickuth, 2008	[29]	retrospective	PrO	Renal cell 18, other 4	Femur 14, humerus 4, other 4	22	22	Complete devascularization vs. incomplete devascularization	Blood loss	1 m, 1 M	No difference in blood loss for various degree of devascularization
6	Kwon, 2010	[30]	retrospective	PrO	Lung 7, renal 4, hepatic 4, other 10	Femur 20, humerus 5	23	25	0	Blood loss	0	Embolization reduces blood loss
7	Koike, 2011	[31]	retrospective	Pa	Hepatic 6, Renal 3, Gynecological 3, Other	Spine 9, pelvis 8, femur 1, rib 1	18	40	0	Pain palliation	4 m	The VAS score was significantly decreased by TACE/TAE.
8	Rossi, 2011	[6]	retrospective	Pa	Renal 84, lung 22, breast 20, other 117	Pelvis 154, spine 83, other 72	243	309	0	Pain palliation	86 m, 1 M	Effective pain palliation was achieved in 97% of procedures, average duration 8.1 months
9	Robial, 2012	[32]	retrospective	PrO	Breast 28, lung 19, renal 16, other 30	Spine	93	35	Embolization vs. no embolization	Blood loss	ND	Embolization reduces blood loss and need for blood replacement
10	Kato, 2013	[33]	retrospective	PrO	Thyroid 39, renal 27	Spine	58	66	Optimal timing between embolization and surgery	Blood loss	0	Embolization reduces blood loss
11	Rossi, 2013	[34]	retrospective	Pa	Renal cell carcinoma	Pelvis 67, spine 32, other 8	107	163	0	Pain palliation	40 m, 1 M	Effective pain palliation was achieved in 96% of procedures, average duration 10 months
12	Pazionis, 2014	[35]	retrospective	PrO	Renal cell carcinoma, thyroid carcinoma	Femur 49, humerus 35, pelvis 31, other 7	118	53	Embolization vs. no embolization	Blood loss, operating time, renal function impairment	2 m	Embolization reduces blood loss and need for blood replacement
13	Clausen, 2015	[36]	RCT	PrO	Lung 17, breast 8, other 20	Spine	45	23	Embolization vs. no embolization	Blood loss, blood replacement, surgery time	4 m, 1 M	Embolization reduces opeative time; blood loss is reduced only in hypervascular metastases
14	Kim, 2015	[37]	retrospective	PrO	HCC	Femur 36, humerus 22, other 17	75	22	Embolization vs. no embolization	Blood loss	ND	Embolization reduces blood loss
15	Ratasvuori, 2016	[38]	retrospective	PrO	Renal cell carcinoma	Femur 82, pelvis 15, other 51	148	56	Embolization vs. no embolization	Blood loss	0	No effect on blood loss after embolization
16	Facchini, 2016	[9]	retrospective	Pa	Renal 54, breast 22, other	Spine	164	178	0	Pain palliation	100 m, 1 M	Effective pain palliation was achieved in 97% of procedures, average duration 9.2 months
17	Jernigan 2018	[39]	retrospective	PrO	Renal cell carcinoma	Femur	1285	135	Embolization vs. no embolization	Transfusion requirements	ND	No effect on transfusion requirements
18	Çelebioğlu, 2021	[40]	retrospective	PrO	Renal cell carcinoma	Pelvis 12, spine 7, other 27	41	46	Optimal timing between embolization and surgery	Blood loss	15 m	Surgery should preferably be performed <1 day after embolization
19	Papalexis, 2023	[41]	retrospective	Pa	Breast, renal, prostate, lung	Sternum	10	14	0	Pain palliation	0	Pain score and analgesic drug consumption were reduced by 50% in all 10 patients (100%, *p* < 0.05).
20	Koob, 2022	[42]	retrospective	PrO	Renal cell carcinoma	Spine 36, extremities 43	54	30	Embolization vs. no embolization	Blood loss, operating time	NA	Embolization of the extremities had a negative effect. No effects on spine.
21	Groot, 2022	[43]	retrospective	PrO	Breast, prostate, lung	Spine	106	53	Embolization vs. no embolization	Blood loss, complications	0	No complications, no benefit from embolization
22	Acuña, 2023	[18]	retrospective	PrO	Renal cell carcinoma 69, thyroid carcinoma 8	Lower extremities 51, upper extremities 25, spine 7	77	46	No embolization vs. embolization < 24 h surgery vs. >24 h surgery	Blood loss	NA	Surgery may be delayed >24 h from embolization. In selected cohorts, embolization may not be needed.

**Table 4:** Overview of clinical applications of arterial embolization for musculoskeletal pathology.

Clinical Setting	Indication	Primary Goals	Key Points
Oncologic	Hypervascular bone metastases (e.g., renal cell carcinoma, thyroid)	Pain palliation, local control	Effective for pain relief and reducing intraoperative blood loss; ideal for hypervascular metastases unresponsive to radio/chemotherapy.
	Primary bone and soft tissue tumors (e.g., aneurysmal bone cysts, desmoid fibromatosis)	Alternative to surgery or percutaneous ablations	Useful when tumors are near neurovascular structures, offering symptom control with minimal invasiveness.
Preoperative	Tumors requiring surgical resection in difficult-to-access areas (e.g., pelvis, spine)	Reduce blood loss during surgery	Reduces perioperative complications and enhances tumor visualization for surgical intervention.
Palliative	Advanced malignancies with limited curative options	Symptom relief	Suitable for patients with poor surgical candidacy, providing temporary symptom relief and improved quality of life.
Degenerative	Knee osteoarthritis, adhesive capsulitis	Pain reduction, functional improvement	Expands options for patients unresponsive to conservative treatments but not candidates for surgery.
Inflammatory Musculoskeletal	Conditions like lateral epicondylitis, chronic tendinopathy	Pain relief, functional recovery	Offers symptom management in chronic inflammatory cases where conservative management has failed.
